# Monitoring Sublethal Injury in *Listeria monocytogenes* During Heat Treatment of Pork Frankfurter-Type Sausages: A Single-Cell vs. Population Level Approach

**DOI:** 10.3390/foods14173144

**Published:** 2025-09-08

**Authors:** Marianna Arvaniti, Eleni Vlachou, Maria Kourteli, Anastasia E. Kapetanakou, Panagiotis N. Skandamis

**Affiliations:** 1 Laboratory of Food Quality Control and Hygiene, Department of Food Science and Human Nutrition, Agricultural University of Athens, Iera Odos 75, 11855 Athens, Greece; marvaniti@aua.gr (M.A.); elenivlachou96@gmail.com (E.V.); marielikourteli@gmail.com (M.K.); pskan@aua.gr (P.N.S.); 2 Institute of Technology of Agricultural Products, Hellenic Agricultural Organization-DIMITRA, S. Venizelou 1, 14123 Lycovrissi, Greece

**Keywords:** *Listeria monocytogenes*, in-package pasteurization, frankfurters, injury, viable-but-not-culturable

## Abstract

*Listeria monocytogenes* is a foodborne pathogen capable of contaminating ready-to-eat meat products, e.g., frankfurters. Post-packaging mild heat treatment via water immersion is commonly employed; however, this may be sublethal to cells located in protected niches or beneath the product surface. The objectives of this study were to evaluate thermal injury of *L. monocytogenes* on frankfurters at single-cell versus population level and to comparatively estimate pathogens’ physiological status. Pork frankfurter-type sausages were inoculated (*ca*. 7.0–7.5 log CFU/cm^2^) with *L. monocytogenes* strain EGDE-e. Heat treatment was performed at 61 °C (*max*. 60 min) and 64 °C (*max*. 12 min). To determine the injured subpopulation from the total, tryptic soy agar with 0.6% yeast extract (TSAYE), supplemented or not with 5% NaCl, was used. Plating-based quantification of injured cells was compared to CFDA/PIstained cells analysed by fluorescence microscopy and quantified with Fiji software. Injury was recorded mainly after 2 and 4 min at 64 °C, whereas no injury was detected at 61 °C, at population level. Following exposure to 61 °C for 60 min, culturable cells dropped below the enumeration limit (0.3 log CFU/cm^2^), while a considerable number of CFDA^+^/PI^−^ and CFDA^+^/PI^+^ cells indicated viable-but-non-culturable induction and sublethal injury, respectively. These findings suggest that non-culturability may limit the accuracy of solely culture-based enumeration methods.

## 1. Introduction

*Listeria monocytogenes* is a widespread foodborne pathogen that poses a significant risk to public health, particularly for ready-to-eat (RTE) products. It can lead to illnesses varying from mild gastrointestinal symptoms to severe invasive listeriosis, a potentially fatal condition for certain high-risk population segments such as children, the elderly, immune-compromised individuals, and pregnant women [1]. According to the European Food Safety Authority (EFSA) Zoonoses Report, in 2022, 27 Member States reported a total of 2738 confirmed cases of invasive *L. monocytogenes* infection, leading to 1330 hospitalizations and 286 deaths. Moreover, the overall case fatality rate across the EU was recorded at 18.1%, marking a notable increase from the respective percentages of 13.7% reported in 2021 and 13.0% in 2020 [2]. Considering the above, the EU has regulated strict safety criteria to limit *L. monocytogenes* in RTE products with which the food industry must comply [3].

A substantial proportion of different types of meat products have been prominently implicated in major human listeriosis outbreaks on a global scale [4]. Processed, vacuum-packaged meat products [5,6], pork tongue [7], sausages [5] and polony [8] were mainly involved in listeriosis outbreaks or sporadic cases. The most extensive recorded listeriosis outbreak in South Africa occurred between 2017 and 2018 and was linked to the consumption of polony, also known as Bologna sausage, a RTE processed meat product [8]. Frankfurters are among the most widely consumed cooked meat products in the RTE category. In fact, according to recent market research data, the global hot dog and sausage market size was valued at USD 82.48 billion in 2023, with projections estimating it will grow to USD 112.41 billion by 2032, reflecting a compound annual growth rate (CAGR) of 3.5% for the forecast period [9]. Listeriosis poses considerable economic implications for the meat industry due to potential product recalls, reputational damage, and the imposition of more stringent regulatory requirements. High-profile outbreaks associated with ready-to-eat (RTE) meat products, including frankfurters, underscore the necessity for rigorous control strategies and continuous surveillance. These measures significantly affect production workflows and may lead to product withdrawals, thus diminishing consumer confidence and reducing market share.

Although adequate cooking stages take place during the production of packaged frankfurters to eliminate the presence of *L. monocytogenes*, there is evidence that the pathogen is often found in the final product [10,11,12]. The main causes may be either a failure in the heating process (with a target temperature above 68.3 °C required to set the emulsion and develop the characteristic colour of the frankfurter) [13] or post-cooking contamination, particularly during the mechanical removal of the cellulose casing used to shape the frankfurters [14,15]. As such, it seems that the application of post-packaging pasteurization is of fundamental importance. In fact, on June 6th of 2003, the United States Department of Agriculture released an interim final rule to encourage processors of RTE products to adopt sanitation procedures and one or more post-lethality treatments (adding antimicrobial ingredients or using processing treatments) to control the pathogen on RTE meat products and poultry [16]. The Australian government has adopted a similar approach, as per the regulatory guidelines released by the Meat Standards Committee (2008). In terms of research, several studies are testing various in-package pasteurization techniques, such as hot water immersion, steam reheating, microwave, ultrasound, high pressure, thermo-sonication, and ohmic heating, which have shown that they may result not only in a safe final product but also in an extended shelf life [17,18,19,20]. However, it seems that immersion in hot water is the most widely used by the RTE meat industry, since it is considered the simplest to be implemented across the product’s processing line.

The most challenging aspect of in-package heat treatment in the food industry is optimising the process parameters (temperature–time combination) to ensure the production of safe ready-to-eat (RTE) products while also preventing undesirable changes in appearance, such as alterations in colour, texture, or the presence of excess fluid. Following this rationale, the application of high temperatures is usually avoided and is being replaced by intermediate temperatures over longer periods. In 2019, the New South Wales Government Food Authority released a food safety schemes manual with detailed guidelines for in-package pasteurization via immersion in hot water for the reduction in *L. monocytogenes* on RTE meats and providing combinations of immersion time and temperature on the meat surface (from 60 °C to >76 °C) to deliver a 6D reduction in the pathogen [21]. Considering the above as prerequisites for ensuring the production of safe and high-quality final RTE meat products, the present study employed two specific temperature-time combinations for the heat treatment of frankfurters, namely 61 °C for *max.* 60 min and 64 °C for *max.* 12 min (see Section 2.2.2). However, it is well-known that under sublethal conditions, bacterial cells can shift through various physiological states from viable to sublethal injury, followed by early dormancy (persisters), progressing to a deeper dormant state (Viable-But-Not-Culturable/VBNC), and finally leading to cell death [22,23]. As Arvaniti and Skandamis [24] noted, injured, persisters, and VBNC cells may elude detection by culture-based ISO methods [25,26], resulting in an underestimation of a product’s actual microbial load. Based on that, the parallel use of single-cell approaches to estimate the physiological heterogeneity of individual cells, especially for persisters and VBNC (early and deep dormancy stage, respectively), may constitute a strategic tool, since they show increased reliability and accuracy in the detection of foodborne pathogens [24] compared to conventional culture-based detection methods, which have low sensitivity [27]. Specifically, previous researchers have studied the viability of VBNC using various fluorescent stains, i.e., acridine orange, carboxy-fluorescein diacetate (CFDA), and propidium iodide (PI), which indicate either cell membrane integrity or enzymatic activity. These stains were combined with fluorescence microscopy or flow cytometry and fluorescence-activated cell sorting, suggesting that these techniques may be quite accurate and promising for assessing food safety of the final product [28,29]. Our research team has also performed extensive research regarding the assessment of sublethal injury and the VBNC state of *L. monocytogenes* induced by various stresses to which cells are commonly exposed along the food chain continuum, i.e., acid stress (acetic acid and HCl; pH 3.0, 2.7, 2.5 at 20 °C for 5 h) or disinfectants (peracetic acid and sodium hypochlorite; 0.5, 5, or 10 ppm at 4 °C and 20 °C) by using staining and fluorescence microscopy [30,31]. However, the existing literature on sublethal injury and the VBNC state of *L. monocytogenes* induced by various stresses is limited to in vitro research.

In light on the above, the objective of this study was to further explore this area by: (i) evaluating in situ the sublethal thermal injury of *L. monocytogenes* on pork frankfurter-type sausages at both single-cell and population level, at surface temperatures (61 °C and 64 °C) representative of in-package hot water immersion treatments and (ii) comparatively analyzing the distribution of viable, sublethally injured, and dead cells of the pathogen during thermal exposure, employing conventional culture-based methods following ISO protocols and fluorescent microscopy.

## 2. Materials and Methods

### 2.1. Microorganism and Inoculum Preparation

The widely used pathogenic *Listeria monocytogenes* strain, EGDe, a food isolate (serotype 1/2a) [32,33], from the microorganism collection of the Laboratory of Food Quality Control and Hygiene in Agricultural University of Athens was used in the present study. Stock cultures were stored at −20 °C in Tryptic Soy Broth (TSB; Oxoid™, Wesel, Germany) supplemented with 0.6% Yeast Extract (YE; Neogen Culture Media, Lansing, MI, USA) (TSBYE) and 20% glycerol. The microorganism was maintained on slants of Tryptic Soy Agar supplemented with 0.6% *w*/*v* Yeast Extract (TSAYE) under refrigeration at 4 °C and was sub-cultured every 3 weeks.

A single colony from the TSAYE stock culture was inoculated into 10 mL TSBYE and incubated for 24 h at 37 °C. After incubation, 2.5 mL of the 24 h culture was aseptically transferred into an Erlenmeyer flask containing 150 mL TSBYE and incubated for 18 h at 37 °C. Cells were collected by centrifugation (2434× *g*; 30 min; 4 °C) (Megafuge 1.0 R; Heraeus, Germany). The pellet was washed twice with ¼ strength Ringer’s solution (Neogen Culture Media, USA) and was finally re-suspended in 10 mL of the same diluent to concentrate bacterial cells. The final population was determined at approximately 10^9^–10^10^ CFU/mL by plating 0.1 mL of the appropriate dilution on a TSAYE plate after incubation for 48 h at 37 °C.

### 2.2. Experimental Procedure

**Figure 1:**
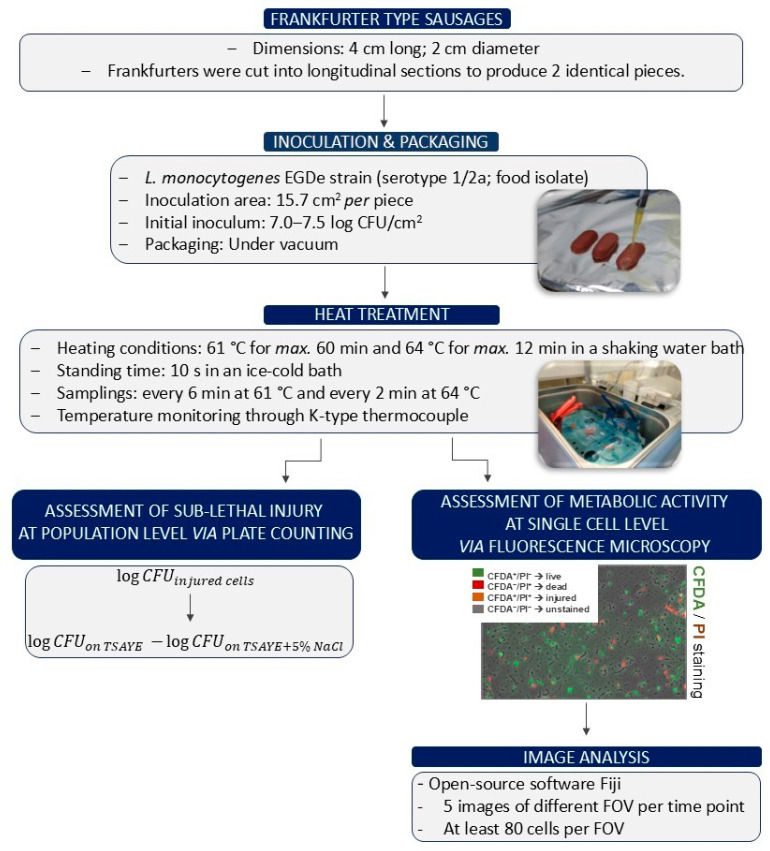
Experimental design.

Figure 1 illustrates the experimental procedure starting from the stages of inoculation and heat treatment of pork frankfurter-type sausages to the assessment of (i) sublethal injury at the population level using the plate count method, and (ii) metabolic activity at the single-cell level through fluorescence microscopy. Three independent experiments were performed, and duplicate samples were used for each trial (n = 6).

#### 2.2.1. Inoculation and Vacuum Packaging of Pork Frankfurter-Type Sausages

Commercial packages of frankfurter-type sausages from pork meat (4 cm long; 2 cm diameter; 220 g per package) were purchased from a local supermarket (Athens, Greece), transferred to the laboratory, and stored at 4 °C for *max*. 2 h. The frankfurters were cut into longitudinal sections to produce 2 identical pieces. Each piece was surface-inoculated by adding 40 µL of inoculum on the outer casing of frankfurter (inoculation area: 15.7 cm^2^) to achieve an initial concentration of *ca*. 7.0–7.5 log CFU/cm^2^ (Figure 1). The initial concentration of the inoculum was chosen to: (i) ensure detectable survivors by the plate counting method after heat treatment, (ii) reduce variability in measurements caused by stochastic survival of individual cells, and (iii) ensure comparability with other published studies. Additionally, as Scott et colleagues reported, the validation of lethal treatments requires higher inoculum levels (5.0–7.0 log CFU/g) [34]. All samples were left at 20 °C for 10 min under aseptic conditions to enhance the attachment of the inoculum. Finally, each frankfurter piece was placed in a plastic bag (dimensions: 15 cm × 10 cm) with gas permeability *ca*. 25, 90, and 6 cm^3^/m^2^ per day/10^5^ Pa for CO_2_, O_2_, and N_2_ at 20 °C and 50% relative humidity (Flexo-Pack S.A., Koropi, Greece) and was vacuum packaged using a sealing packaging machine (Lerica C312-CHEF, Via dell’Artigianato, 30, 30024 Musile di Piave, Italy).

#### 2.2.2. Heat Treatment of Pork Frankfurter-Type Sausages

Frankfurter sausages were heat-treated at 61 °C and 64 °C in a shaking water bath (Stuart SBS40, Bibby Sterilin Ltd., Staffordshire, UK) to ensure a more rapid come-up time and more homogeneous temperature distribution (Figure 1). The temperature of water and frankfurters (non-inoculated sample) was externally monitored by using two different thermocouples (TC-08 Data Logger; Pico Technology Ltd., St. Neots, UK). Specifically, the temperature of the frankfurter was recorded by embedding the tip of the thermocouple approximately 1–2 mm beneath the surface to ensure its stable positioning throughout the heating treatment. At the same time, the sample was subsequently vacuum sealed as it is described in Section 2.2.1. As time 0 was defined, the point at which the surface temperature of the sausage reached the target temperature. Inoculated non-heated samples were used as controls to verify the initial inoculum. The selected combinations of heating time and temperature were based on the recommended process time-temperature of RTE meats’ surface (ranging from 60 °C to > 76 °C) outlined in the food safety schemes manual for in-package pasteurization using hot water immersion, which aims to achieve a 6D reduction in L. *monocytogenes* [21]. Specifically, heat treatment was performed at 61 °C and 64 °C, with samplings taken every 6 min for *max.* 60 min at 61 °C and every 2 min for *max*. 12 min at 64 °C, respectively. At each sampling point, two packages were removed from the heated water bath and cooled immediately in an ice-water bath (*ca*. 0–2 °C) for 10 s to achieve temperature equilibration (final temperature of samples < 25–30 °C) and possible reduction in cold and hot spots. Three independent experiments were performed, and duplicate samples were used for each trial (n = 6).

#### 2.2.3. Assessment of Sublethal Injury at Population Level via Plate Counting

The exterior of each package was disinfected by spraying 70% ethanol and subsequently opened under aseptic conditions. Aliquots of 3 mL of Ringer’s isotonic solution (recovery volume) were added to each package and were massaged by hand for about 3 min, while subsequently, serial decimal dilutions were prepared in ¼ strength Ringer’s solution. To differentiate the sublethal injured cells from the total culturable population, selective and non-selective media, namely TSAYE and TSAYE supplemented with 5% NaCl (*w*/*v*) (AppliChem GmbH, Darmstadt, Germany), respectively, were used. Siderakou et al. [35] showed that 5% NaCl is the maximum non-inhibitory salt concentration for *L. monocytogenes,* in which only the healthy cells may increase due to reduced resistance of injured cells. Based on the above, aliquots of 100 µL from the appropriate dilution were surface plated on TSAYE at 37 °C for 48 h and on TSAYE + 5% NaCl after incubation at 37 °C for 5 days. The enumeration limit was 0.3 log CFU/cm^2^ calculated by the following equation:

(1) $$Enumeration\;limit=\frac{\left(CFU\times 10^{dilution\;factor+1}\right)\times Recovery\;volume}{Inoculated\;Area}$$

Thus, sublethal injury at each sampling point during thermal stress was determined by using the following equation [35]:

(2) $$\log{CFU}_{injured\;cells}=\log{CFU}_{on\;TSAYE}-\log{CFU}_{on\;TSAYE+5\%\;NaCl}$$

#### 2.2.4. Assessment of Metabolic Activity at Single-Cell Level via Fluorescence Microscopy

The assessment of metabolic activity at single-cell level via fluorescence microscopy was carried out after exposure for 0, 6, 12, 18, 60 min at 61 °C or 0, 2, 4, 6 min at 64 °C. All time points corresponding to population-level decrease were selected for sampling to investigate the physiological state of the cells during these intervals, except for the 60 min point at 61 °C. This time point was included in the sampling plan to assess the physiological state of cells when the culturable population was below the detection limit (0.3 log CFU/cm^2^). At each sampling point, 100 µL of the homogenate (Section 2.2.3) was added in 1 mL sterile Eppendorf and was stained in a final concentration of 10 μM CFDA (Sigma-Aldrich; Burlington, MA, USA) in dimethyl sulfoxide (DMSO) and 29.92 μM PI solution in DMSO (LIVE/DEAD™ BacLight™ bacterial viability kit, Thermo Fisher Scientific; Waltham, MA, USA). Cells were kept at 20 °C in the absence of light for 20 min. From LIVE/DEAD^TM^ BacLight^TM^ bacterial viability kit, only PI was used. Simultaneous CFDA/PI staining was conducted.

CFDA stains bacteria that possess active esterases and intact membranes, signalling metabolic activity through green fluorescence, whereas PI selectively enters cells with compromised membranes, indicative of cell death, and emits red fluorescence. To discard residues of fluorophores, cells were centrifuged (13,800× *g*, 2 min) and resuspended in 20 µL of Ringer’s solution. Untreated cells from the same culture as the heat-treated cells were centrifuged (2434× *g*; 10 min; 4 °C) and resuspended in 3 mL of sterile Ringer’s solution. These cultures were utilized to prepare both positive and negative controls. For the positive controls, 100 mL of untreated cells in mid-stationary-growth-phase were suspended in sterile Ringer’s solution and stained with CFDA. To generate negative controls, 100 mL amounts of the aforementioned cultures were centrifuged (13,800× *g*; 2 min) and resuspended in 70% ethanol for 30 min at 20 °C. The ethanol-treated (killed) cells were subsequently centrifuged (13,800× *g*; 2 min) and resuspended in 100 mL sterile Ringer’s solution and stained with PI.

Assessment of cell’s staining status revealed 4 distinct fractions of cells: (i) viable cells exhibiting metabolic activity with intact membranes (CFDA^+^/PI^−^), (ii) dead cells with compromised membranes (CFDA^−^/PI^+^), (iii) sublethally injured cells displaying metabolic activity despite membrane damage (CFDA^+^/PI^+^) and (iv) cells lacking esterase activity but maintaining membrane integrity (CFDA^−^/PI^−^).

Fold of change (FC) between the mean percentage of treated cells at each time point (MPT) and the mean percentage of cells at time zero (MPT_0_) was calculated for CFDA^+^PI^−^ (viable), CFDA^−^PI^+^ (dead), CFDA^+^PI^+^ (sublethally injured), and CFDA^−^PI^−^ (non-esterase active with membrane integrity) subpopulations according to the protocol used by [36,37]. Specifically, the fold of change was determined using the following formulas:

(3) $$FC\left({CFDA}^{+}{PI}^{-}\right)=\frac{MPT\left({{CFDA}^{+}{PI}^{-}}_{stressed\;cells\;at\;each\;time\;point}\right)}{MPTZ\left({{CFDA}^{+}{PI}^{-}}_{time\;zero}\right)}$$

(4) $$FC\left({CFDA}^{-}{PI}^{+}\right)=\frac{MPT\left({{CFDA}^{-}{PI}^{+}}_{stressed\;cells\;at\;each\;time\;point}\right)}{MPTZ\left({{CFDA}^{-}{PI}^{+}}_{time\;zero}\right)}$$

(5) $$FC\left({CFDA}^{+}{PI}^{+}\right)=\frac{MPT\left({{CFDA}^{+}{PI}^{+}}_{stressed\;cells\;at\;each\;time\;point}\right)}{MPTZ\left({{CFDA}^{-}{PI}^{-}}_{time\;zero}\right)}$$

(6) $$FC\left({CFDA}^{-}{PI}^{-}\right)=\frac{MPT\left({{CFDA}^{-}{PI}^{-}}_{stressed\;cells\;at\;each\;time\;point}\right)}{MPTZ\left({{CFDA}^{-}{PI}^{-}}_{time\;zero}\right)}$$

Fold change (FC) values below 1 indicate a reduction in the evaluated subpopulation relative to the control condition.

The same clonal cell population was used for both plate counting and fluorescence microscopy *per* biological replicate. For each sampling point, 5 distinct fields of view (FOV) were analysed, with each FOV containing a minimum of 80 cells. Fluorescence microscopy images were analysed using the open-source software Fiji (version 2.16.0; https://fiji.sc/, accessed on 5 February 2025) [38].

Fluorescent microscopy experiments were performed using an inverted fluorescence microscope (Leica DMi8) with a DFC 7000T camera (Leica; Wetzler, Germany) and LAS X software (Leica; Wetzler, Germany). Cells were observed with an oil immersion 100 phase-contrast objective with a numeric aperture value of 1.25.

### 2.3. Statistical Analysis

To assess statistically significant differences in the mean *L. monocytogenes* population (log CFU/cm^2^) across different heat treatment time points, a one-way analysis of variance (ANOVA) followed by Tukey’s Honestly Significant Difference (HSD) post hoc test was conducted. Differences were considered statistically significant at a 95% confidence level (*p* < 0.05). A two-way ANOVA was performed between the common sampling time points (0, 6, and 12 min) to analyse the treatment temperature and exposure time effect on *L. monocytogenes* population (log CFU/cm^2^). Chi-squared test [39] was performed to compare the percentages of stressed *L. monocytogenes* cell subpopulations (CFDA^+^/PI^−^, CFDA^−^/PI^+^, CFDA^+^/PI^+^, and CFDA^−^/PI^−^) at different exposure times. Further, statistical analysis of cell subpopulations (raw cell counts) was performed using multinomial logistic regression to evaluate the effect of exposure time on the distribution of fluorescence categories (CFDA^+^/PI^−^, CFDA^−^/PI^+^, CFDA^+^/PI^+^, with CFDA^−^/PI^−^ being used as the reference condition). Independent replicates at each time point were included in the analysis. Odds ratios (OR) with 95% confidence intervals (CI) were calculated for each category relative to the reference group. All analyses were conducted in R (version 4.2.2) using the VGAM package. Forest plots were generated to visualize the odds ratios and confidence intervals for each subpopulation over time. Statistical analyses were performed in R [40].

## 3. Results

### 3.1. Enhanced Survival of Resistant Subpopulations as Reflected by the Reported Tailing of Inactivation Curves

**Figure 2:**
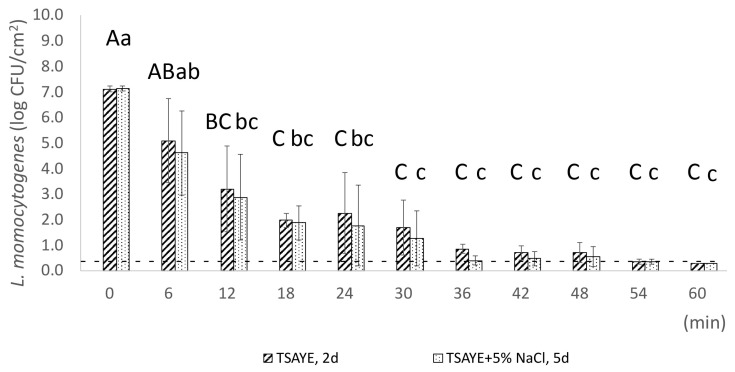
Populations of *L. monocytogenes* strain EGDe (log CFU/cm^2^) were measured following heat treatment at 61 °C for *max.* 60 min. Bars represent mean values ± SD from three independent biological replicates. Statistical differences in the mean *L. monocytogenes* population across time points were assessed using one-way ANOVA followed by Tukey’s Honestly Significant Difference (HSD) test. Statistically significant differences (*p* < 0.05, CI 95%) are indicated by different uppercase letters (for TSAYE) and lowercase letters (for TSAYE + 5% NaCl). The dotted line denotes the detection limit (0.3 log CFU/cm^2^).

**Figure 3:**
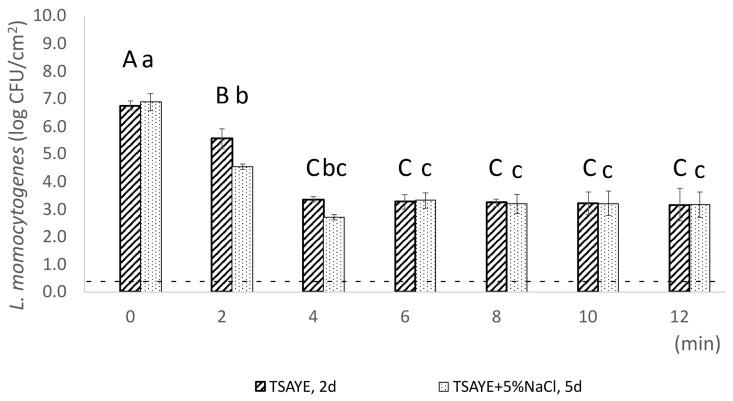
Populations of *L. monocytogenes* strain EGDe (log CFU/cm^2^) were measured following heat treatment at 64 °C for *max.* 12 min. Bars represent mean values ± SD from three independent biological replicates. Statistical differences in the mean *L. monocytogenes* population across time points were assessed using one-way ANOVA followed by Tukey’s Honestly Significant Difference (HSD) test. Statistically significant differences (*p* < 0.05, CI 95%) are indicated by different uppercase letters (for TSAYE) and lowercase letters (for TSAYE + 5% NaCl). The dotted line denotes the detection limit (0.3 log CFU/cm^2^).

Heat treatment of pork frankfurter samples was evaluated at two different temperatures, 61 °C (every 6 min) for *max*. 60 min and 64 °C (every 2 min) for *max*. 12 min, respectively (Figure 2 and Figure 3). A two-way ANOVA was performed for the common sampling time points (0, 6, and 12 min) to analyse the heat treatment temperature and the exposure time effect on *L. monocytogenes* population (log CFU/cm^2^). Statistical analysis showed that there was no significant interaction between treatment temperature and exposure time on *L. monocytogenes* population (log CFU/cm^2^) (*p* = 0.34; Table 1). However, exposure time alone showed a statistically significant effect on the enumerated population, as indicated by a *p*-value of 0.0004 (Table 1). These results indicate that the reduction in *L. monocytogenes* population is primarily associated with time rather than temperature or their interaction. The strong effect of time (with a significant F-value) suggests that prolonged exposure is the dominant factor driving population reduction. Meanwhile, the modest 3 °C temperature increase from 61 to 64 °C did not produce a statistically significant effect, consistent with the expected log-linear relationship between temperature and inactivation rate. According to the principles of thermal inactivation, an increase of 3 °C in temperature is expected to have a significant impact on microbial population reduction. However, as Monu et colleagues have reported, the z-values of different strains of *L. monocytogenes* ranged from 4.16 to 6.59 °C [41], meaning that a 3 °C increase represents less than a one-log change in the D-value. Consequently, the observed reduction in bacterial counts is predominantly driven by exposure time rather than by this relatively small temperature increment.

Evaluation of sublethal injury at population level, using plate counting, resulted in the enumeration of the total culturable (enumerated on TSAYE), the non-injured (enumerated on TSAYE + 5% NaCl), and the sublethally injured population (Equation (2)). The enumerated total culturable population of *L. monocytogenes* (on TSAYE) on frankfurters significantly decreased (*p* < 0.05) from 7.1 log CFU/cm^2^ (SD: ±0.1 log CFU/cm^2^) to 3.2 log CFU/cm^2^ (SD: ±1.7 log CFU/cm^2^) after exposure to 61 °C for 12 min (Figure 2), while TSAYE population remained ca. 2.0 log CFU/cm^2^ between the 18th and 30th min of heat treatment. Moreover, it is notable that a tailing was observed between 36 and 60 min at 61 °C, indicating heterogeneity and robustness among individual cells toward sublethal heat exposure (Figure 2). However, in terms of statistics, the enumerated total culturable population of samples exposed to 61 °C from 18 to 60 min remained stable (*p* > 0.05). According to previous studies, tailing, as a phenomenon, upon exposure to different lethal stresses, i.e., lethal heat, HHP, and low pH conditions, has been observed in several foodborne pathogens like *L. monocytogenes*, *Salmonella* Typhimurium, and *Escherichia coli* O157:H7 [42,43,44,45,46,47]. The key feature of this well-reported response (tailing) is that the initial exponential inactivation is followed by a slower decrease, which has been linked to reduced cell sensitivity due to heterogeneity in microbial population [48], as well as genotypic and phenotypic diversity [49]. At single-cell level, tailing is reflected in the persistence of CFDA^+^/PI^−^ cells, which remained at a high percentage of 46.8% after exposure to 61 °C for 60 min. Sublethal injury was not detected following heat treatment at 61 °C, which may be attributed to the high variability observed in the surviving *L. monocytogenes* population, particularly at 6 and 12 min of heat exposure. Increased variability after heat treatment of *L. innocua* at 61 °C has also been reported by Aguirre and colleagues [50]. Specifically, their findings showed a marked increase in variability when the population of survivors fell below 1 and revealed that inactivation times were heterogeneously distributed across the population, indicating the presence of subpopulations with varying heat resistance [50].

Induction of sublethal injury at population level was recorded after 2 min (1.0 log CFU/cm^2^) and 4 min (0.6 log CFU/cm^2^) of heat exposure at 64 °C (Figure 3). Sublethal injury is an intermediate physiological state in the dormancy continuum, where cells may present damage in non-critical components and/or in critical components, but at low intensity [51], after exposure to one or more sublethal treatments [52]. Contrary to 61 °C, our results indicate that sublethal injury was induced at population level during heat treatment at 64 °C for 2 and 4 min, potentially due to the higher temperature. Similarly to our results, Wang and colleagues reported that increasing treatment temperature from 60 °C to 65 °C increased the proportion of sublethally injured *L. monocytogenes* cells on agar surface [53]. In fact, previous researchers have reported that the severity of sublethal damage depends on the intensity and the duration of the treatment [54]. Sublethal cellular injury may be reversible through intrinsic repair mechanisms or may progress to irreversible damage, ultimately culminating in cell death [54]. Bacterial injury manifests differently by temperature because repair processes require energy and protein synthesis, which are optimized at or near a bacterium’s optimal growth temperature, typically around 35–37 °C [55,56]. Therefore, temperatures near a bacterium’s optimal growth temperature facilitate the efficient repair of heat-induced cellular damage [55,57]. While higher temperatures cause heat damage by altering the structure of cellular macromolecules, this damage may be sublethal and thus reversed if bacterial cells are subsequently exposed to the appropriate conditions for repair. Tailing has also been detected in the inactivation curve of 64 °C between 6th and 12th min of exposure, reflecting the existence of a fraction of cells with increased heat resistance.

### 3.2. Induction of Sublethal Injury and VBNC State at Single-Cell Level

**Figure 4:**
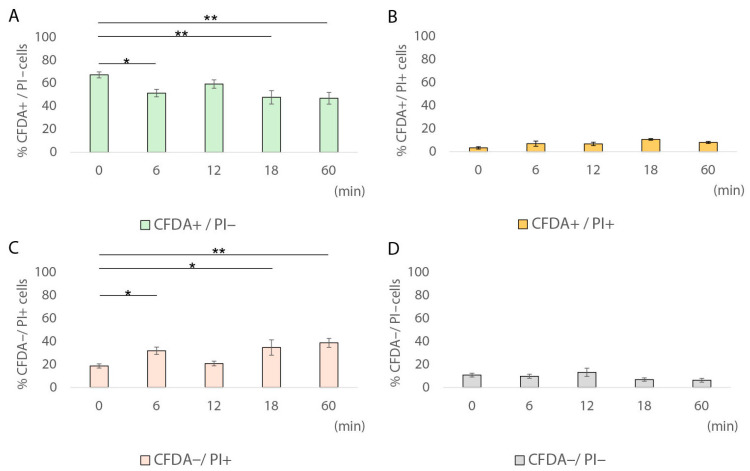
Percentages of *L. monocytogenes* EGDe cell subpopulations after heat treatment at 61 °C for 0, 6, 12, 18, and 60 min. Bars represent mean values ± SE from 5 distinct fields of view (FOV), with each FOV containing a minimum of 80 cells. Chi-squared test was performed to compare the percentages of stressed *L. monocytogenes* cell subpopulations [green bars, CFDA^+^/PI^−^, (**A**); orange bars, CFDA^+^/PI^+^, (**B**); red bars, CFDA^−^/PI^+^, (**C**), and grey bars, CFDA^−^/PI^−^, (**D**)] at different exposure times. *, *p* < 0.05; **, *p* < 0.01.

During the 60 min heat treatment of pork frankfurter-type sausages at 61 °C, sublethally injured cells (CFDA^+^/PI^+^) were detected only at single-cell level, ranging from 6.9% to 10.6% (Figure 4B), whereas they were not detected at population level (Figure 2). Our results showed that after 18 min at 61 °C, the fraction of sublethally injured cells increased by 3.2 times compared to time 0 (10.6% vs. 3.3%; Table 2). On the contrary, after 60 min of heat treatment, the percentage of CFDA^+^/PI^−^ cells had significantly decreased compared to time 0; however, it remained relatively high (46.8%, SD: ±11.4; *p <* 0.01). Under the same conditions (61 °C for 60 min), the entire population (total culturable and non-injured population) was below the detection limit (0.3 log CFU/cm^2^). The latter result indicates the existence of a fraction of cells that remained viable (CFDA^+^/PI^−^), but they were non-culturable, thus potentially indicating the induction of the VBNC state in *L. monocytogenes* under the specified conditions. Several previous studies have reported the induction of *Listeria* spp. into the VBNC state [30,31,36,37,58,59,60]. In fact, VBNC induction has been associated with exposure to various stress factors such as starvation, salinity, visible light, inoculum size, weak acids, disinfectants, and temperature [30,37,61,62]. Specifically, Besnard and colleagues showed that temperature is one of the physicochemical factors that play a crucial role in VBNC induction in *L. monocytogenes* [62]. In detail, for all the tested *L. monocytogenes* strains, except for the Scott A strain, exposure to 20 °C rapidly triggered VBNC induction, while lower temperatures (4 °C) did not have the same effect [62]. The results of our study indicate that the VBNC state may potentially be induced in situ in *L. monocytogenes* during the heat treatment of frankfurters at sublethal temperatures. However, the resuscitation capacity of this cell fraction requires further experimental verification. Regarding the membrane-damaged–dead cells (CFDA^–^/PI^+^), their percentage gradually increased during exposure at 61 °C from 31.85% at 6 min to 38.75% at 60 min (*p* > 0.05). Finally, the percentage of non-esterase active with membrane integrity cells (CFDA^−^/PI^−^) did not show any statistically significant changes (*p* > 0.05) during exposure at 61 °C compared to time 0.

**Figure 5:**
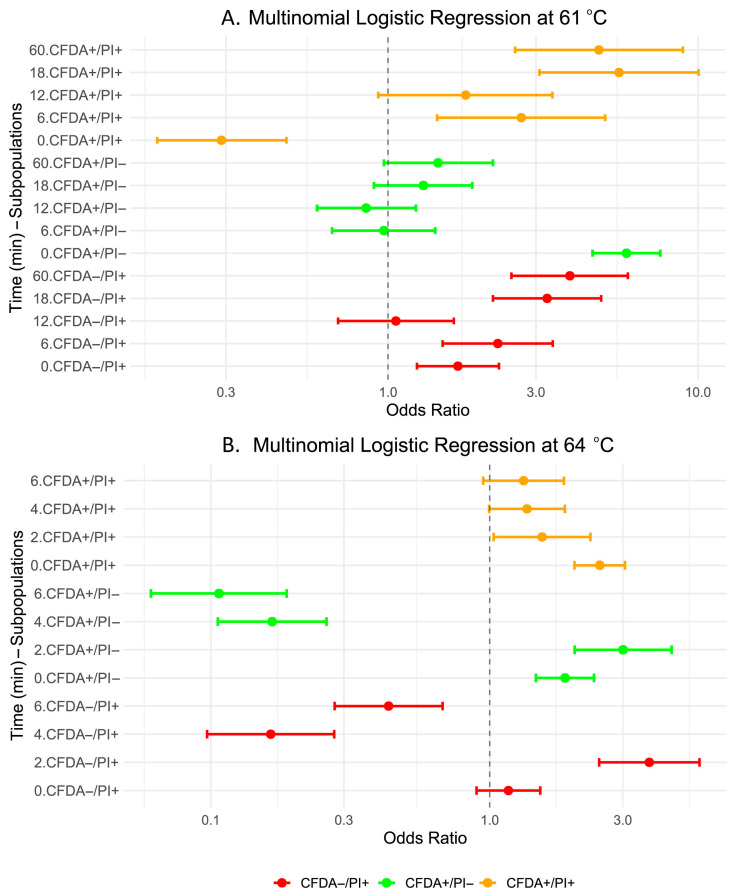
Multinomial logistic regression of cell subpopulations (green, CFDA^+^/PI^−^; red, CFDA^−^/PI^+^; and orange, CFDA^+^/PI^+^) at different heat treatment conditions. Forest plots depict odds ratios (OR) with 95% confidence intervals for each cell subpopulation compared to the reference group (CFDA^−^/PI^−^) at different time points (in minutes). Panel A shows results at 61 °C, and Panel B at 64 °C. The vertical dashed line at OR = 1 represents no difference from the reference category.

Multinomial logistic regression analysis was also performed to assess changes in *L. monocytogenes* EGDe cell subpopulations after heat treatment at 61 °C for up to 60 min, using the CFDA^–^/PI^−^ population as the reference category (Table 3; Figure 5A). At baseline (0 min of exposure), the odds ratios of viable (CFDA^+^/PI^−^) and dead cells (CFDA^−^/PI^+^) were significantly higher compared to the reference condition, CFDA^−^/PI^−^ (OR = 5.88, 95% CI: 4.57–7.56, *p* < 0.001; OR = 1.68, 95% CI: 1.24–2.28, *p* < 0.001), whereas the odds ratio of sublethally injured (CFDA^+^/PI^+^) cells was significantly lower (OR = 0.29, 95% CI: 0.18–0.47, *p* < 0.001). After 6 min of heat treatment, dead (CFDA^−^/PI^+^) and sublethally injured (CFDA^+^/PI^+^) fractions of cells exhibited significant increases in odds ratios relative to the reference (OR = 2.26, 95% CI: 1.50–3.40, *p* < 0.001; OR = 2.69, 95% CI: 1.44–5.02, *p* = 0.002), while metabolically active (CFDA^+^/PI^−^) cells odds ratio remained comparable to the reference condition (*p* > 0.05). From 18th to 60th min of exposure, membrane-damaged–dead (CFDA^−^/PI^+^) and injured cells (CFDA^+^/PI^+^) showed a marked increase in odds ratios compared to the reference condition, with ORs of 3.26 and 5.56 at 18 min and 3.86 and 4.79 at 60 min, respectively (*p* < 0.001). On the contrary, metabolically active-viable (CFDA^+^/PI^−^) cells’ odds ratio showed no significant differences from the reference condition from the 6th until the 60th min of heat treatment (*p* > 0.05). These findings indicate a progressive shift toward membrane-damaged–dead (CFDA^–^/PI^+^) and sublethally injured (CFDA^+^/PI^+^) subpopulations as the time of exposure increased. Furthermore, they confirm tailing at single-cell level, which is reflected in the persistence of CFDA^+^/PI^−^ cells.

**Figure 6:**
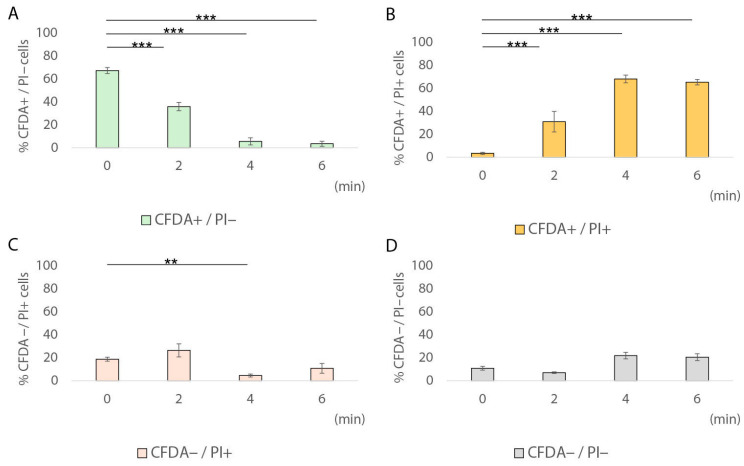
Percentages of *L. monocytogenes* EGDe cell subpopulations after heat treatment at 64 °C for 0, 2, 4, and 6 min. Bars represent mean values ± SE from 5 distinct fields of view (FOV), with each FOV containing a minimum of 80 cells. Chi-squared test was performed to compare the percentages of stressed *L. monocytogenes* cell subpopulations [green bars, CFDA^+^/PI^−^, (**A**); orange bars, CFDA^+^/PI^+^, (**B**); red bars, CFDA^−^/PI^+^, (**C**), and grey bars, CFDA^−^/PI^−^, (**D**)] at different exposure times. **, *p* < 0.01; ***, *p* < 0.001.

Regarding treatment of pork frankfurter-type sausages samples at 64 °C, the observed induction of sublethal injury at population level was also confirmed microscopically, at single-cell level (Figure 6B). Specifically, after 2 and 4 min of exposure at 64 °C, the percentage of CFDA^+^/PI^+^ cells was *ca*. 9-fold (30.9% vs. 3.3%; *p <* 0.001) and 21-fold (68.1% vs. 3.3%; *p <* 0.001) higher compared to time 0, respectively. Interestingly, a significantly high percentage of sublethally injured cells (65.3%, SD: ± 5.2; *p <* 0.001) was detected at single-cell level after 6 min at 64 °C compared to time 0 at the same treatment conditions (Figure 5B). This fraction of sublethally injured cells was not detected at population level, using plate counting method, indicating that single-cell techniques, such as fluorescence microscopy coupled with CFDA/PI staining, may broaden our ability to reveal the extent of physiological heterogeneity of bacterial cells [24] (Figure 3). Regarding metabolically active—viable cells (CFDA^+^/PI^−^), their percentage significantly decreased from 35.8%, after 2 min of exposure at 64 °C, to 5.6% and 3.5%, after 4 and 6 min of exposure, respectively (*p <* 0.001) (Figure 6A), while the quantified fraction of dead cells showed a pick (26.4%) after 2 min of sample’s treatment at 64 °C (Figure 6C).

Multinomial logistic regression analysis revealed significant shifts in the distribution of *L. monocytogenes* EGDe subpopulations during heat treatment at 64 °C, using CFDA^−^/PI^−^ as the reference condition (Table 4; Figure 5B). At baseline (0 min), metabolically active (CFDA^+^/PI^−^) cells had 1.86-fold higher odds ratio (95% CI: 1.47–2.37, *p* < 0.001), while sublethally injured (CFDA^+^/PI^+^) cells had 2.48-fold higher odds ratio (95% CI: 2.01–3.06, *p* < 0.001) compared to the reference condition. At the same time, the odds ratio of dead (CFDA^–^/PI^+^) cells did not differ significantly (*p* = 0.253). After 2 min of heat treatment at 64 °C, the odds ratios for viable (CFDA^+^/PI^−^) and dead (CFDA^−^/PI^+^) cells increased significantly to 3.01 (95% CI: 2.02–4.49, *p* < 0.001) and 3.73 (95% CI: 2.47–5.65, *p* < 0.001), respectively, while sublethally injured (CFDA^+^/PI^+^) cells odds ratio remained moderately elevated (OR = 1.54, 95% CI: 1.03–2.30, *p* = 0.034). Prolonged heating, for 4 min at 64 °C, caused a pronounced reduction in the odds ratios of metabolically active and dead fractions of cells, with ORs of 0.17 (95% CI: 0.11–0.26, *p* < 0.001) and 0.16 (95% CI: 0.10–0.28, *p* < 0.001), respectively, and further slight decreases until the 6th min of heat treatment (ORs = 0.11 and 0.43, both *p* < 0.001). In contrast, the odds ratio of CFDA^+^/PI^+^ subpopulation decreased more gradually.

A comparison of the 6 min exposure between the two heat treatment temperatures revealed a clear shift in the physiological state of *L. monocytogenes* cells as the temperature increased from 61 °C to 64 °C (Figure 4 and Figure 6). At 61 °C, over half of the cells (51.5%, SD: ±6.6) remained CFDA^+^/PI^−^, indicating metabolic activity and membrane integrity (Figure 4A), while only 3.3% of cells retained the status of viable at 64 °C (Figure 6A). Conversely, the proportion of sublethally injured cells (CFDA^+^/PI^+^) increased significantly from 6.9% at 61 °C to 65.3% at 64 °C after 6 min of exposure, indicating that higher heat exposure caused extensive membrane damage while still preserving esterase activity in a large portion of the population (Figure 6B). The fraction of dead cells (CFDA^−^/PI^+^) decreased from 31.8% at 61 °C to 10.7% at 64 °C, likely reflecting a shift toward sublethal injury rather than complete inactivation or cell death (Figure 6C). Notably, the CFDA^−^/PI^−^ subpopulation, potentially representing dormant, sublethally injured, or VBNC cells, increased from 9.8% to 20.4% when the exposure temperature was higher, suggesting that elevated heat stress may induce a non-esterase active with membrane integrity fraction of cells (Figure 6D). The hypothesis that unstained cells (CFDA^−^/PI^−^) represent a non-culturable subpopulation with membrane integrity has previously been reported in *L. monocytogenes* AA-treated cells [31]. It is plausible that the fraction of CFDA^−^/PI^−^ cells that lack esterase activity while maintaining membrane integrity potentially indicates a dormant physiological state characterized by markedly reduced metabolic activity or a transient phase of sublethal injury.

### 3.3. Transition of Metabolically Active Cells (CFDA^+^/PI^−^) to Sublethally Injured Cells (CFDA^+^/PI^+^) at Single-Cell Level

A transition of metabolically active cells (CFDA^+^/PI^−^) to sublethally injured cells (CFDA^+^/PI^+^) was detected as heat exposure temperature increased from 61 °C to 64 °C, at single-cell level (Figure 4B and Figure 6B). During thermal exposure at 61 °C, two fractions of cells were dominant in the total population, namely the viable (CFDA^+^/PI^−^) and the dead (CFDA^−^/PI^+^) cells, at all exposure times (Figure 4A,D). In detail, the ratio of viable cells decreased, while simultaneously the ratio of dead cells increased during exposure (Table 2). Increasing the heat treatment temperature from 61 °C to 64 °C resulted in a shift in the detected fractions of the total population from viable (CFDA^+^/PI^−^) observed at earlier exposure times (2 min), to sublethally injured cells (CFDA^+^/PI^+^) detected at later stages of heat treatment (6 min). Induction of sublethal injury was also detected at the population level after thermal exposure to 64 °C, mainly after 2 and 4 min of exposure (Figure 3). Consequently, the ratio of sublethally injured cells increased during exposure to 64 °C, indicating that cells entered an intermediate physiological state before death. Further experiments are needed to decipher the mechanisms underlying this shift. Sublethal injury may be temporary and then repaired by the cellular machinery, or may evolve into severe permanent damage, leading to cellular death [54]. There is a correlation between the intensity of stress, the resulting physiological states of the cells, and their capacity for resuscitation [24]. As environmental conditions change and may become more favourable, bacterial cells can resuscitate in response to a stimulus. Resuscitation of dormant pathogenic cells can be induced by various factors, including elevated nutrient availability, temperature fluctuations, chemical stimuli, or co-cultivation with host cells [63]. The latter suggests that post-heat treatment conditions of frankfurters, such as transportation or storage conditions, may play a crucial role in the resuscitation of this fraction of cells.

## 4. Conclusions

The findings of this study highlight the influence of heat treatment temperature on the physiological state of *L. monocytogenes* cells in situ (on the surface of pork frankfurter-type sausages). At population level, the observed tailing in the inactivation curves at both 61 °C and 64 °C suggests heterogeneity in thermal resistance, reflecting the enhanced survival of resistant subpopulations. At single-cell level, exposure to 61 °C resulted in cells that were below the enumeration limit yet retained viability (CFDA^+^/PI^−^), potentially indicating the induction of the VBNC state. However, to further confirm the induction of the VBNC state, additional resuscitation experiments should be conducted in future investigations. In addition, the detection of CFDA^+^/PI^+^ cells highlights the presence of sublethally injured cells at single-cell level. Microscopic analysis following heat treatment at 64 °C further confirmed the induction of sublethal injury. Notably, as the exposure temperature increased from 61 °C to 64 °C, a transition from metabolically active (CFDA^+^/PI^−^) to sublethally injured cells (CFDA^+^/PI^+^) was observed, indicating a shift toward sublethal injury rather than complete inactivation or cell death. These results highlight the complexity of bacterial inactivation under heat stress, as well as the need to consider the cell’s physiological state as population heterogeneity before designing and performing thermal processing strategies. One approach to evaluate the impact of heat stress on bacterial cells at single-cell level is by monitoring the lag times of individual cells that successfully recover. Several studies have demonstrated substantial variation in lag times among single-cells within a population, with this variability increasing in response to more severe heat treatments [64,65,66]. Understanding how heat treatments influence single-cell lag time variability is critical for accurately assessing the risk of recovery and subsequent growth of stressed pathogenic bacteria in processed foods, where low numbers of such cells may be unevenly distributed across different product units [65]. Another important aspect is that conventional culture-based detection methods exhibit limited sensitivity due to the occurrence of VBNC cells [27]. Nevertheless, reference methods in food microbiology are internationally standardized by the International Organization for Standardization (ISO), and most rely on conventional, culture-dependent, population-level microbiological approaches. To address current limitations, validation procedures incorporating novel single-cell detection and monitoring techniques, i.e., fluorescence microscopy coupled with CFDA/PI staining, are required [24]. Ideally, culture-based population methods and single-cell strategies should be applied in parallel to accurately assess the bacterial heterogeneity and dormancy phenomena [24]. Evaluation of *L. monocytogenes* sub-lethal injury and dormancy phenomena in situ could be crucial for food microbiology, as it provides new insights into the risks associated with overestimating process lethality.

## Figures and Tables

**Table 1:** Two-way Anova table for *L. monocytogenes* population (log CFU/cm^2^) as a function of treatment temperature and exposure time. (***, *p* < 0.001).

	Df	F Value	Pr (>F)	Sig.
Time	2	21.17	<0.001	***
Temperature	1	2.21	0.17	
Time: Temperature	2	1.22	0.34	
Residuals	9			

**Table 2:** Fold change (FC) between the mean percentage of treated at each time point (MPT) and the mean percentage of time zero (MPT_0_) calculated for each of the detected subpopulations, i.e., CFDA^+^/PI^−^ (metabolically active with membrane integrity-viable), CFDA^−^/PI^+^ (membrane damaged-dead), CFDA^+^/PI^+^ (metabolically active with damaged membranes-sublethally injured), and CFDA^−^/PI^−^ (non-esterase active with membrane integrity). Values below 1 indicate a decrease in the evaluated subpopulation relative to the control condition.

Temperature	Exposure Time (min)	FC CFDA^+^/PI^−^	FC CFDA^−^/PI^+^	FC CFDA^+^/PI^+^	FC CFDA^−^/PI^−^
61 °C	6	0.76	1.71	2.10	0.91
	12	0.88	1.11	2.04	1.22
	18	0.71	1.86	3.22	0.64
	60	0.70	2.08	2.42	0.58
64 °C	2	0.53	1.41	9.37	0.64
	4	0.08	0.24	20.67	2.03
	6	0.05	0.58	19.82	1.90

**Table 3:** Odds ratios of *L. monocytogenes* EGDe cell subpopulations after heat treatment at 61 °C for 0, 6, 12, 18, and 60 min. The table shows the results of a multinomial logistic regression using CFDA^−^/PI^−^ as the reference category. Odds ratios greater than 1 indicate increased odds relative to CFDA^−^/PI^−^, whereas values less than 1 indicate decreased odds. Time 0 represents the baseline measurement. **, *p* < 0.01; ***, *p* < 0.001.

Category	Time	Odds Ratio	Lower 95% CI	Upper 95% CI	*p*-Value	Signif.
CFDA^+^/PI^−^	0	5.88	4.57	7.56	<2 × 10^−16^	***
CFDA^+^/PI^−^	6	0.97	0.66	1.42	0.852103	
CFDA^+^/PI^−^	12	0.85	0.59	1.23	0.398458	
CFDA^+^/PI^−^	18	1.30	0.90	1.87	0.164295	
CFDA^+^/PI^−^	60	1.45	0.97	2.18	0.070375	
CFDA^−^/PI^+^	0	1.68	1.24	2.28	0.000746	***
CFDA^−^/PI^+^	6	2.26	1.50	3.40	9.82 × 10^−5^	***
CFDA^−^/PI^+^	12	1.06	0.69	1.63	0.804948	
CFDA^−^/PI^+^	18	3.26	2.18	4.87	9.24 × 10^−9^	***
CFDA^−^/PI^+^	60	3.86	2.50	5.94	1.34 × 10^−9^	***
CFDA^+^/PI^+^	0	0.29	0.18	0.47	1.50 × 10^−6^	***
CFDA^+^/PI^+^	6	2.69	1.44	5.02	0.001892	**
CFDA^+^/PI^+^	12	1.78	0.93	3.39	0.081872	
CFDA^+^/PI^+^	18	5.56	3.08	10.04	1.14 × 10^−8^	***
CFDA^+^/PI^+^	60	4.79	2.57	8.94	1.12 × 10^−6^	***

**Table 4:** Odds ratios of *L. monocytogenes* EGDe cell subpopulations after heat treatment at 64 °C for 0, 2, 4, and 6 min. The table shows the results of a multinomial logistic regression using CFDA^−^/PI^−^ as the reference category. Odds ratios greater than 1 indicate increased odds relative to CFDA^−^/PI^−^, whereas values less than 1 indicate decreased odds. Time 0 represents the baseline measurement. *, *p* < 0.05; ***, *p* < 0.001.

Category	Time	OR	Lower 95% CI	Upper 95% CI	*p*-Value	Signif.
CFDA^+^/PI^−^	0	1.86	1.47	2.37	4.03 × 10^−7^	***
CFDA^+^/PI^−^	2	3.01	2.02	4.49	5.99 × 10^−8^	***
CFDA^+^/PI^−^	4	0.17	0.11	0.26	8.46 × 10^−15^	***
CFDA^+^/PI^−^	6	0.11	0.06	0.19	2.45 × 10^−14^	***
CFDA^−^/PI^+^	0	1.17	0.90	1.52	0.253	
CFDA^−^/PI^+^	2	3.73	2.47	5.65	6.68 × 10^−10^	***
CFDA^−^/PI^+^	4	0.16	0.10	0.28	3.76 × 10^−11^	***
CFDA^−^/PI^+^	6	0.43	0.28	0.68	0.000293	***
CFDA^+^/PI^+^	0	2.48	2.01	3.06	9.54 × 10^−15^	***
CFDA^+^/PI^+^	2	1.54	1.03	2.30	0.0344	*
CFDA^+^/PI^+^	4	1.36	1.00	1.86	0.0545	
CFDA^+^/PI^+^	6	1.32	0.95	1.85	0.101	

## Data Availability

The original contributions presented in this study are included in the article. Further inquiries can be directed to the corresponding author.
